# A cost comparison of open versus percutaneous approaches to management of large staghorn calculi

**DOI:** 10.4103/0970-1591.38599

**Published:** 2008

**Authors:** Maneesh Sinha, K. R. John, K. N. Chacko, Ganesh Gopalakrishnan

**Affiliations:** Department of Urology, Christian Medical College, Vellore, India; 1 The Clinical Epidemiology Unit, Christian Medical College, Vellore, India

**Keywords:** Open surgery, percutaneous nephrolithotripsy, staghorn calculi

## Abstract

**Aim:**

This paper compares the cost of open versus percutaneous approaches to the management of large staghorn calculi in a tertiary care hospital in India.

**Materials and Methods:**

Patients who underwent surgery for staghorn calculi larger than 6 cm between January 1998 and December 2003 were included. Those who had confounding factors in terms of cost such as additional surgical or medical procedures and complications unrelated to the surgery were excluded. The process of costing was done by following the clinical pathway.

**Results:**

There were 13 patients who had open stone surgery and 19 patients who underwent percutaneous nephrolithotripsy (PCNL). The major differences in cost were seen in the higher cost of instruments and consumables in the PCNL group. The cost of management of complications widened this gap. Two patients in the PCNL group and none in the residual group required redo surgery. The residual stones in the open and PCNL groups required a mean of 2525 and 3623 shocks per patient respectively. Complete clearance after redo surgery and Shockwave lithotripsy (SWL) was seen in 92% and 58% in the open and PCNL arms respectively. The overall cost per patient was $625 per PCNL and $499 per open surgery. The final mean residual stone size in the PCNL group was 4.84 mm whereas it was 0.38 mm in the open group. The effective cost of achieving complete clearance in one patient was $1078 in the PCNL group and $543 in the open group.

**Conclusion:**

Open stone surgery is less costly than PCNL in large staghorn calculi.

## INTRODUCTION

We are living in an age where the seductive charm of new technology often influences management decisions. However, we also live in a country where cost is invariably of overriding concern. In a situation where there are different treatment options available the issue of the cost of open surgery in comparison to modern surgical approaches comes into focus. One such situation is a large staghorn calculus. The importance of this issue is not limited to our institution. All urologists in India are fund managers trying to make limited budgets meet unlimited demands. While the benefits of PCNL over open stone surgery are well established this paper compares the procedures purely in terms of direct costs.

### Aim

This paper compares the cost of open versus percutaneous approaches to the management of large staghorn calculi.

## MATERIALS AND METHODS

### Assumptions

While costing the procedures some assumptions were made. A period of five years was taken as the time in which the capital costs were to be earned back. This was done keeping in mind that technological advancements were likely to render current instruments obsolete in this period. The cost of services outsourced from the department was taken at face value i.e. the rate at which it was charged to the patient. This included laboratory services, radiological procedures and anesthesia.

### Demographic data

The study was carried out between January 1998 and December 2003. All adults with staghorn stones larger than 6 cm were included. Patients who underwent additional surgical procedures unrelated to the stone, those who required additional expenses in view of medical co-morbidities, children and those patients who had complications that were not attributable to the surgical procedure were excluded. These were all considered to be confounding factors in the cost analysis.

In the actual costing the clinical pathway was followed, i.e. the patients were followed from the time they presented to the outpatient till they were discharged and the costs involved at each stage were computed. At each stage direct costs to the hospital e.g. cost of purchase of disposables, salaries were calculated. Hospital accounts of individual patients were not referred to. The decision to proceed with open or percutaneous surgery was that of the individual consultants keeping in focus the likely cost and the patient's ability to pay for treatment. The demographic characteristics, clearance rates and requirement of additional procedures were compared and accounted for.

The total costs incurred in each group were divided by the number of patients in each group and the mean cost was taken as representative of a single patient. The representative patients were then compared in terms of cost.

### Formulae

#### Personnel

The cost of personnel was computed by adding up the annual expenses on salary, accommodation as well as the perks of each class of personnel. This was divided by the number of minutes of work per year.

#### Per day cost

One day's stay in the ward was computed by incorporating the cost of doctors, nurses, infrastructure and actual disposables and drugs used.

#### Cost per shock

Analyzing the capital costs as well as recurring costs on the lithotripter during the study period compared the cost of lithotripsy. Other than replacement of parts this included the expenses on staff, electricity, linen, drugs and the annual maintenance contracts. Using the actual number of shocks used during the study period gave us the recurring cost per shock. The cost of lithotripsy for residual stones in the representative patients was calculated by multiplying the cost per shock by the mean number of shocks in each group.

### Instruments

Instruments used for PCNL were divided into three groups. The first comprised those instruments which could be used only in PCNL. The second included those which could be used in all kinds of endoscopic stone surgery. The last group included those which could be used in all endoscopic procedures. The replacements for endoscopic instruments during the study period were also divided into similar groups. The cost of instruments per case in each group was computed by dividing the total cost in each group by the number of the respective procedures done during the study period. Adding up the cost per case in each group and dividing this by the number of PCNLs done during the study period derived the cost of instruments for each PCNL.

Open surgical instruments were divided into general instruments and instruments which were used only in open stone surgery. The recurring costs were similarly classified. Using the number of procedures in each group as the denominator the cost of instruments per case was computed.

### Outsourcing

Services that were outsourced from other departments were taken at face value. This was a valid decision keeping in mind that the department pays for these at the same rate for patients who undergo free treatment. Biochemical, hematological and radiological investigations, anesthesia and intensive care charges, the cost of blood and blood products as well as the charges of angioinfarction were included in this group.

### Drugs and disposables

All the drugs and other consumables used in each group were recorded and the mean cost per patient was derived.

### Overheads

The departments that earn revenue pay towards the cost of running the non-earning departments. This adds to the recurring cost of the department and this cost has been incorporated.

### Infrastructure

The infrastructure cost was calculated by using existing rental rates and or by applying a depreciation of 5% per year to construction costs. This was translated into cost per patient by using the number of patients per day as the denominator.

### Currency conversion

All values in Indian Rupees were converted to US dollars with the exchange rate of Rs. 43.67 per US dollar.

## RESULTS

### Demographic data

There were 13 patients in the open group and 19 patients in the PCNL group. In the open group two patients had extended pyelolithotomy, two had anatrophic nephrolithotomy while all the others had pyelolithotomy with radial nephrotomies. The median stone size was 7.25 cm (range 6 - 10.2 cm) in the open group and 7.5 cm (range 6.2 - 9.8 cm) in the PCNL arm. Open surgery lasted for a mean of 240 min whereas PCNL lasted for 201 min. Open surgery achieved clearance rates of 61.5%. Complete clearance was recorded in 26.3% in the PCNL arm. Four patients in the open group versus two in the PCNL arm had hemorrhage necessitating blood transfusion. The mean blood requirement per patient was 1 unit in the open arm and .89 units in the PCNL group. One patient underwent angioinfarction after PCNL. The other major complications encountered were sepsis (one in each group) and hydrothorax (two in the PCNL arm) (Appendix Table 1). Two patients in the PCNL arm and none in the open group needed redo surgery. Four and 10 patients in the open and PCNL groups respectively underwent lithotripsy. The mean shocks per patient during lithotripsy for residual stones were 2525 in the open group and 3623 in the PCNL arm. After redo surgery and ESWL complete clearance rates improved to 58% in the PCNL arm. After ESWL for the residual stones in the open surgery group complete clearance was seen in 92% of patients. The final mean residual stone size after lithotripsy and redo surgery was .38 mm in the open arm and 4.84 mm in the PCNL group.

**Appendix Table 1:** Complications

Complications	Open	PCNL
Major		
Hemorrhage	4	2
Sepsis	1	1
Hydrothorax	0	2
Minor		
Fever	6	9
Wound infection	1	0
Pleural rent	1	0

### Cost of personnel

The annual expense on each category of staff was totaled. The salary cost of accommodation as per market rates, provident fund expenses, actual health expenditure on staff and staff dependents were totaled. The number of minutes put in by the personnel was computed. After accounting for leave and weekends there are 282 working days or 40.29 weeks every year in our institution. In one week each member works for 48 h (Appendix Table 2).

**Appendix Table 2:** Cost of personnel

	Salary and perks	Accommodation	Provident fund	Health benefits	Total	Total min of work	Cost $/min
Professor	6957	2200	696	225	10,077	116035	0.087
Registrar	1925	825	0	225	2976	116035	0.026
Staff nurse	1787	0	179	225	2191	116035	0.019
Radiographer	1870	0	187	225	2282	116035	0.020
Attender	1375	0	138	225	1737	116035	0.015
Sweeper	1217	0	122	225	1564	116035	0.014

### The clinical pathway

**Figure 1:**
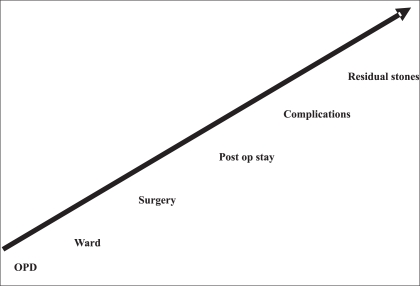
Clinical Pathway

The costs of the patients as they presented to the outpatient department (OPD) and then progressed toward final discharge are presented [Figure 1].

### 1. OPD

All patients presenting to the OPD were given a similar set of investigations. The cost of personnel and infrastructure involved was added to the cost of investigations. This totaled to $75.01 in both groups (Appendix Table 3).

**Appendix Table 3:** Outpatient department cost

Investigations	73.00
Doctor	0.39
Supporting staff	0.08
Infrastructure	1.54
Total	75.01

### 2. Ward (preoperative)

Once the patient was admitted in the ward he was seen by the urology as well as the anesthesia registrars. Nurses, ward boys and sweepers were present round the clock. The cost of drugs that were spent on the representative patients was added in each group. Three units of blood were cross-matched for all open cases. In the PCNL group each patient had only one unit cross-matched. The infrastructure cost was again included. Here the cost was slightly higher in the open group ($16.68 for open versus $13.82 for PCNL) (Appendix Table 4).

**Appendix Table 4:** Preoperative costs

	Time spent	Open	PCNL
Urology registrar	15 min	0.38	0.38
Anesthesia registrar	15 min	0.38	0.38
Urology consultant	2 min	0.17	0.17
Staff nurse	24 hrs	5.36	5.36
Drugs		1.94	1.94
Cross-match		6.53	2.18
Infrastructure		1.91	1.91
Total		16.68	13.82

### 3. Surgery

The various variables involved in the actual surgery were computed separately to give the cost per patient [Table 1].

**Table 1:** Overall cost of surgery

	Open	PCNL
Anesthesia	64.54	50.84
Instruments	35.68	109
Personnel	35.40	25.32
Consumables	67.30	75.60
Image intensifier	27.15	40.90
Total	230.07	301.66

#### a. Anesthesia

Anesthesia was charged at a rate of $9.05 per hour. To this was added the mean cost of actual anesthetic drugs. The mean duration of anesthesia was 277 min in the open group and 231 min in the PCNL group. The cost of anesthetic drugs was also higher in the open group (Appendix Table 5).

**Appendix Table 5:** Anesthesia charges

	Open	PCNL
Charge per patient	41.83	34.90
Drugs	22.71	15.95
Total	64.54	50.84

#### b. Instruments

Our department has two sets of PCNL instruments. The cost of instruments used only for PCNLs including all replacements during the study period was $50,335. Five hundred and eighty PCNLs were done during this period. The costs of instruments used in endoscopic stone surgery and all endoscopic procedures were $9118 and $41,944 respectively. Therefore the cost of instruments used for PCNLs per case was $102.48. One thousand two hundred and eighty endoscopic stone surgeries and 4893 endoscopic surgeries were done in this period respectively. These instruments are sterilized using glutaraldehyde. The expense on glutaraldehyde divided by the total number of endoscopic procedures resulted in a cost of $6.71 per endoscopic procedure. Therefore the cost of instruments calculated by adding the cost per case in each of these groups was $109/PCNL.

We have four sets of instruments that are used for a wide variety of major open urological procedures. The total cost of these sets including recurring costs over five years is $39,928. A set of 11 stone-holding forceps priced at $189 is used only in open stone surgery. One thousand three hundred and seven major procedures were done using these sets. One hundred and ninety-three open stone surgeries were done. The charge paid for sterilizing this set is $4.17. The cost of instruments is 35.68 per open stone surgery (Appendix Table 6).

**Appendix Table 6:** Expense on instruments

	Cost	No. of procedures	Cost/case	Sterillization/case	Total
PCNL only	50,335	580	86.78		
Endoscopic stone surgery	9118	1280	7.13		
All endoscpic surgery	41,944	4893	8.57		
				6.71	109
Open stone	189	193	0.96		
All open major	39,928	1307	30.55		
				4.17	35.68

#### c. Consumables

The consumables included catheters, guide wires, sutures and the cost per case in the open and PCNL groups was $67.30 and $75.60 respectively.

#### d. Image intensifier

The image intensifier was required in both groups. This was outsourced from radiology and was charged at $27.15 for open surgery and $40.90 for PCNL.

#### e. Personnel

The operating surgeon in these cases is always a senior consultant. One registrar scrubs in a PCNL whereas two are required in an open procedure. Both procedures require one floor and one scrub nurse. For the endoscopic instruments an instrument technician is employed who spends about 20 min per PCNL in cleaning and replacing instruments after surgery. The mean duration of surgery was 240 min in the open arm and 201 min in the PCNL arm. Using the previously computed per minute rate for personnel the cost in the open and PCNL groups comes to $35.40 and $25.32 respectively.

Thus, the total cost of the surgical procedure was $230.06 in the open group and $301.83 in the PCNL arm (Appendix Table 2).

### 4. Postoperative stay

Postoperative stay was accounted as the cost of mean number of days of stay as well as the cost of drugs. The mean stay in patients without major complications was 7.5 days in the open group and 7.36 in the PCNL group. The cost per day in the ward included the cost of personnel, housekeeping and infrastructure and amounted to $7.49/day. The cost of drugs and other consumables in the open group was $48.72 and it was $44.41 in the PCNL arm. Thus the total cost of uncomplicated postoperative stay in the open and PCNL groups was $104.93 and $99.56 respectively (Appendix Table 7).

**Appendix Table 7:** Cost of postoperative stay

	Days of stay	Cost/day ($)	Days X (cost/day)	Cost of drugs ($)	Total ($)
Open	7.5	7.49	56.21	48.72	104.93
PCNL	7.36	7.49	55.16	44.41	99.56

### 5. Complications

The complications in each group were recorded. These were also converted into a mean cost per patient by computing the additional days of stay per patient and the consumables.

The additional stay due to complications was 1.5 days per patient in the open group and 2.5 per patient in the PCNL group. One patient in the PCNL group required three days in the ICU. Another required angioinfarction. These costs were converted into a mean cost per patient and added to the cost in the representative patient. This totaled to $18.97 in the open group and $53.32 in the PCNL group (Appendix Table 8).

**Appendix Table 8:** Cost of complications

	Cost of additional days of stay	Angioinfarction	ICU	Consumables and investigations	Total
PCNL	2.5 × 7.49 = 18.73	15.67	5.20	13.70	53.32
Open	1.5 × 7.49 = 11.24	0	0	7.72	18.97

### 6. Residual stones

Most patients with residual stones underwent lithotripsy. Two patients in the PCNL group and none in the open arm required redo surgery. Redo surgeries were again followed along the clinical pathway and costs per PCNL evaluated. This came to $12.6/PCNL. The annual recurring expenditure on the lithotripter was divided by the mean shocks per year in the study period to give the cost per shock (Appendix Table 9).

**Appendix Table 9:** Cost per shock during lithotripsy

	Annual expenditures	Shocks/year	Cost per shock
Annual maintenance contract	1,4230.00	2954185	$0.021
Replacement of parts	12,946.00		
Electricity	3849.67		
Staff	9010.70		
Overheads	3437.21		
Sundry	229.15		
Rent	4582.95		
Machine	13748.80		
Other equipment	108.85		

Two thousand two hundred and twenty-five shocks were required in the open group per patient and 3263 in the PCNL group. Thus the total cost of residual stones in the PCNL group was $69.52 + 12.60 = $82.12. In the open group it was $53.80

### Final cost

**Figure 2:**
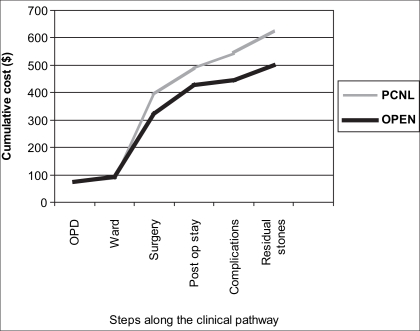
Cumulative costs along the clinical pathway (USD)

The final cost per patient was $499 in the open group and $625 in the PCN group [Table 2 and Figure 2].

**Table 2:** Overall cost ($)

Cost	Open	PCNL
Outpatient department	75.01	75.01
Preoperative	16.68	13.82
Surgery	230.07	301.66
Post-op stay	104.93	99.56
Complications	18.97	53.32
Residual stones	53.80	82.12
Total	499.46	625.49

Effective treatment in stone disease is defined as complete clearance. The cost-effectiveness of each procedure was calculated using the formula, [cost per patient/percentage of patients achieving complete clearance]. The effective cost of achieving complete clearance in one patient was $1078.43 in the PCNL arm and $542.89 in the open arm.

## DISCUSSION

The first question that needs to be addressed is whether open stone surgery is still valid. As early as 1994, The Nephrolithiasis Guidelines Panel of the American Urological Association had said that for all staghorn calculi a combination of percutaneous stone removal and shock wave lithotripsy should be used.[1] The issue of cost was not discussed. Current urological literature continues to support open stone surgery as a valid option in complex large staghorn calculi.[2–6] We consider cost to be of major importance and have analyzed the issue of open versus endoscopic approaches primarily in terms of cost. In addition to a urological perspective this paper includes the active contribution of a health economist (KRJ) as an author. As the benefits of a reasonably straightforward PCNL over open stone surgery are well established we have analyzed this issue only for extremely large stones using 6 cm as an arbitrary cutoff.

Clearance rates for combination therapy (PCNL plus ESWL) in staghorn calculi have been reported as ranging from 59-84%.[7–11] In our PCNL group complete clearance was seen in 26.3% after PCNL alone and this improved to 58% after ESWL to residual calculi. These clearances are explained by the fact that we have included only very large staghorns. Stone-free rates for open surgery range from 65-100%.[2–4,12,13] In our study stone-free rate after open surgery was 61.5% and this improved to 92% after lithotripsy.

An earlier study from India has stated a significantly higher cost for combination therapy as compared to open surgery.[11] The comparison of cost, however, was not the primary objective of that study. In our paper we have compared and quantified the differences in the direct medical costs of open and endoscopic approaches to large staghorn calculi. Open stone surgery was more economical and more efficacious in terms of stone clearance.

It needs to be mentioned here that this study is not a cost-benefit analysis. It limits itself to cost analysis. The benefits of PCNL are irrefutable. Although this study is a retrospective analysis and has a small sample size it does suggest that open stone surgery may be a financially more feasible option in the largest of large staghorn stones.

A very desirable objective would be to reduce the cost of PCNL. In our study the major contributors to the excess cost of PCNL were the expenses on instruments, complications and residual stones. Interestingly, if the capital costs on instruments alone are removed from consideration PCNL still remains a more expensive procedure ($516 *vs*. $463). However, if in addition we assume that both procedures could be done without complications and with complete stone clearance in all patients the PCNL actually becomes slightly less expensive than open stone surgery ($381 *vs*. $391)! The obvious implication is that where PCNL can achieve complete clearance with minimal complications it is financially competitive with open stone surgery. Further improvement in expertise would make PCNL cost-effective even in large stones.

We would like to re-emphasize that individual costs were computed at each step during the clinical pathway. Hospital bills were not referred to as these would not have reflected true costs. We found the clinical pathway to be a useful tool while computing costs for these two surgical procedures.

## CONCLUSIONS

This article is limited in its sample size as well as its scope. It compares the two available surgical modalities purely in terms of cost and is not a cost-benefit analysis. However, it does highlight an aspect of the treatment of complex stone disease which does not seem to have been studied before. The International Consultation on Stone Disease has recognized economics of the treatment of stone disease to be an important consideration. Complications in surgery are significantly reduced with subspecialization. This is true of endourology where the safety margin is low and the percutaneous treatment of staghorn calculi in particular. Costs of treatment escalate when complications arise. The object of this article is not to disprove the value of PCNL in the treatment of stone disease. However, in developing nations, where costs are borne by patients and governments and partly by doctors offering services at concessional rates, we need to understand what exactly all this amounts to. We also need to remember that irrespective of the developments in the treatments of stone disease, we have not been able to reduce the recurrence rates. Hence it would seem logical that when cost considerations alone are discussed, then open stone surgery is less costly. Our data does warrant caution in attempting PCNL in large complex stones where cost is of overriding concern.

## References

[1] Segura JW, Preminger GM, Assimos DG, Dretler SP, Kahn RI, Lingeman JE (1994). Nephrolithiasis Clinical Guidelines Panel summary report on the management of staghorn calculi. J Urol.

[2] Joual A, Dassouli B, Hafiani M (2000). Bivalve anatrophic nephrolithotomy. Ann Urol (Paris).

[3] Melissourgos ND, Davilas EN, Fragoulis A, Kiminas E, Farmakis A (2002). Modified anatrophic nephrolithotomy for complete staghorn calculus disease - Does it still have a place?. Scand J Urol Nephrol.

[4] Sy FY, Wong MY, Foo KT (1999). Current indications for open stone surgery in Singapore. Ann Acad Med Singapore.

[5] Matlaga BR, Assimos DG (2002). Changing indications of open stone surgery. Urology.

[6] Kane CJ, Bolton DM, Stoller ML (1995). Current indications for open stone surgery in an endourology center. Urology.

[7] Pearle MS, Nakada SY, Womack JS, Kryger JV (1998). Outcomes of contemporary percutaneous nephrostolithotomy in morbidly obese patients. J Urol.

[8] Wong MY (1998). Evolving technique of percutaneous nephrolithotomy in a developing country: Singapore General Hospital experience. J Endourol.

[9] Rodrigues Netto N, Claro Jde A, Ferreira U (1994). Is percutaneous monotherapy for staghorn calculus still indicated in the era of extracorporeal shockwave lithotripsy?. J Endourol.

[10] Gerber GS (1999). Combination therapy in the treatment of patients with staghorn calculi. Tech Urol.

[11] Goel MC, Ahlawat R, Bhandari M (1999). Management of staghorn calculus: analysis of combination therapy and open surgery. Urol Int.

[12] Androulakakis PA, Michael V, Polychronopoulou S, Aghioutantis C (1990). Evaluation of open surgery for staghorn calculi in children. Child Nephrol Urol.

[13] Zargooshi J (2001). Open stone surgery in children: Is it justified in the era of minimally invasive therapies?. BJU Int.

